# Dynamic classification of fetal heart rates by hierarchical Dirichlet process mixture models

**DOI:** 10.1371/journal.pone.0185417

**Published:** 2017-09-27

**Authors:** Kezi Yu, J. Gerald Quirk, Petar M. Djurić

**Affiliations:** 1 Department of Electrical and Computer Engineering, Stony Brook University, Stony Brook, NY, United States of America; 2 Department Of Obstetrics, Gynecology and Reproductive Medicine, Stony Brook University, Stony Brook, NY, United States of America; University of Minnesota, UNITED STATES

## Abstract

In this paper, we propose an application of non-parametric Bayesian (NPB) models for classification of fetal heart rate (FHR) recordings. More specifically, we propose models that are used to differentiate between FHR recordings that are from fetuses with or without adverse outcomes. In our work, we rely on models based on hierarchical Dirichlet processes (HDP) and the Chinese restaurant process with finite capacity (CRFC). Two mixture models were inferred from real recordings, one that represents healthy and another, non-healthy fetuses. The models were then used to classify new recordings and provide the probability of the fetus being healthy. First, we compared the classification performance of the HDP models with that of support vector machines on real data and concluded that the HDP models achieved better performance. Then we demonstrated the use of mixture models based on CRFC for dynamic classification of the performance of (FHR) recordings in a real-time setting.

## Introduction

Fetal heart rate (FHR), along with other physiological signals, is routinely monitored before and during labor to assess fetal health. The first fetal monitor became commercially available in 1968 [1], and ever since, electronic fetal monitoring (EFM) has been widely used in hospitals in the U.S. Its rate of use in obstetric practice has climbed from 68.4% in 1989 to 85.2% in 2002 [2].

Nowadays, the evaluation of FHR signals is primarily performed visually by experienced physicians, following guidelines published by various medical institutions including the National Institute of Child Health and Human Development (NICHD) [3] and the International Federation of Gynecology and Obstetrics (FIGO) [4]. These guidelines define different patterns of FHR, such as baseline, variability, acceleration and deceleration. Based on the combination of appearances of certain patterns, the FHR tracings are classified into three classes, “normal”, “indeterminate” and “abnormal.”

Notwithstanding the long presence of FHR tracings in obstetrics, their use for assessing the well-being of fetuses has constantly been questioned. For example, in a recent study, it has been reported that the subjective assessment of FHR tracings exhibits large inter- and intra-variability [5]. The study has also shown that the sensitivity of clinicians’ majority vote to objective outcomes was only 39%. This and similar findings suggest that the high false positive rates have led to increase in the rate of cesarean section deliveries [6], which altogether have put the benefits of using EFM under criticism [7].

The deficiencies of subjective assessment of FHRs raise the need for modern and computerized methods for their processing. Such methods would be able to provide objective and consistent evaluation and to capture hidden dynamics in FHR signals, which are often too challenging for human’s eyes’ inspection. Furthermore, machine learning techniques have been proved to be extremely successful in real-world applications in various fields in recent years. The advances in machine learning have been also reflected in research on FHR classification. This research has produced a number of newly proposed computerized methods.

In one approach, in addition to morphological features proposed in the guidelines [3], for quantifying the underlying patterns of FHR, advanced features extraction algorithms were applied. In [8], the authors worked with several linear and nonlinear features. The former included short-term and long-term variabilities whereas the latter were features related to power spectra and entropy. In [9], a more comprehensive collection of non-linear features were used to model the non-linearity of FHR signals. These features included fractal dimension, approximate entropy, sample entropy, and the Lempel Ziv complexity. The classification of the features was carried out by a support vector machine (SVM) algorithm.

SVMs have not been in the only machine learning methodology for classification of FHR signals. In [10], artificial neural networks were employed as the classifier with 6 FHR features and 6 clinical variables as inputs. Two types of generative models, naïve Bayes and hidden Markov models, were implemented in [11], which were novel attempts because the majority of the methods in the literature were based on discriminative models. In [12], the authors explored the performance of linear regression and SVMs with different kernels. This work also included feature selection and the use of reduction methods such as random forest and principle component analysis (PCA).

The search for better solutions based on machine learning algorithms with more flexibility and robustness as well as better overall performance has continued. Hierarchical Dirichlet process (HDP) mixture models [13], for instance, free the classic mixture models from fixing the number of mixing components, and allow for modeling of grouped data jointly. These models exhibit excellent performance in areas such as information retrieval and topic modeling [14].

In this paper, for classifying FHR signals, we propose two novel approaches based on HDP. We describe the underlying principles of the approaches and show on real-world data that they have very good performance in terms of accuracy and probabilistic interpretations of the results.

The paper is organized as follows. First, we provide some background on non-parametric Bayesian statistical models used in the paper, and we discuss their advantages over traditional parametric models (Hierarchical Dirichlet Process Mixture Models). Then, in the subsequent section, Features for Classification, we describe the set of features we used in our experiments. The details of our experimental setting, including the database, the pre-processing of the data, the dimensionality reduction of the feature space, and the performance assessment are explained in the section Experimental Settings. Results obtained by our approach and comparisons with an existing method are provided in the section Results. In the last section, we discuss the results and make final conclusions.

The main contribution of this paper lies in the novelty in applying hierarchical Dirichlet process-based mixture models to FHR classification. In our previous research, we explored a set of suitable features for our models and obtained several preliminary results with HDP mixture models [15, 16]. Here, we continue to explore the potential of the models and present classification results with probabilistic interpretations. An important extension of our work is the use of the time-varying models from [17] to achieve dynamic real-time classification.

## Hierarchical Dirichlet process mixture models

In this section, we describe the two mixture models that we implemented in our experiments, the well-known HDP mixture model and our time-varying modification of it. We explain how one can generate data by using the models and then how one can conduct inference about the model unknowns from data that are generated by the model.

### Notation

In the problems of our interest, the observations are organized into groups. We adopt the notation from [13], where *x*_*j*,*i*_ denotes the *i*th observation in the *j*th group. We consider that each observation is drawn independently from a mixture model. In the context of FHR problems, each group **x**_*j*_ = (*x*_*j*,1_, *x*_*j*,2_, …) corresponds to features of one FHR recording, and each observation *x*_*j*,*i*_ to features of one segment of the recording.

### Models

We start with the HDP mixture models proposed in [13] and then describe their modification proposed in [17] to accommodate for time-evolving statistics of the data.

#### Hierarchical Dirichlet process mixture models

A hierarchical Dirichlet process defines a set of random probability measures *G*_*j*_ linked to a global random probability measure *G*_0_. Specifically, *G*_0_ is distributed as a Dirichlet process (DP) with concentration parameter *γ* and base probability measure *H*, i.e.,

$$\begin{array}{c} G_{0}|\gamma ,H∼\text{DP}\left(\gamma ,H\right). \end{array}$$ (1)

The random measures *G*_*j*_’s are conditionally independent given *G*_0_, and distributed according to

$$\begin{array}{c} G_{j}|\alpha ,G_{0}∼\text{DP}\left(\alpha ,G_{0}\right), \end{array}$$ (2)

where *α* is also a concentration parameter. We explain this model and its extension to mixture models by way of the Chinese restaurant franchise (CRF) metaphor.

Suppose that there is a restaurant franchise with a shared menu across the restaurants. With *x*_*j*,*i*_ we denote the *i*th customer in the *j*th restaurant, and with *θ*_*j*,*i*_, the *dish type* served to this customer. In this setup, the customers correspond to the observations *x*_*j*,*i*_, a restaurant corresponds to an FHR recording **x**_*j*_ and the dish type to a parameter set of a distribution used for drawing the observations. The index *z*_*j*,*i*_ is the index of such parameter set and associated with the observation *x*_*j*,*i*_.

Next, we introduce *K* iid random variables *ϕ*_1_, …, *ϕ*_*K*_, which represent global dishes and which are distributed according to *H*. Each customer *x*_*j*,*i*_ is seated at a table, denoted by *t*_*j*,*i*_, and each table is paired with one dish *ϕ*_*k*_. Furthermore, let *ψ*_*j*,*t*_ represent the dish served on table *t* in restaurant *j*, and *k*_*j*,*t*_ be the indicator of the dish served on table *t* in restaurant *j*. For example, *t*_3,4_ = 6 means that customer 4 in restaurant 3 sits at table 6, *ψ*_3,6_ = *ϕ*_*k*_3,6__ signifies that on table 6 in restaurant 3 dish *ϕ*_*k*_3,6__ is served, where *k*_3,6_ ∈ {1, 2, ⋯, *K*}. With this notation, we have that *θ*_3,4_ = *ϕ*_*z*_3,4__, where *z*_3,4_ ∈ {1, 2, ⋯, *K*}. Note the difference between *k*_*j*,*t*_ and *z*_*j*,*i*_. The former is the index of the dish served in restaurant *j* on table *t*, and the latter, the index of the dish served to customer *i* in restaurant *j*.

We also need a notation for counts. With *n*_*j*,*t*,*k*_ we denote the number of customers in restaurant *j* at table *t* serving dish *k*, and with *m*_*j*,*k*_, the number of tables in restaurant *j* serving dish *k*. We represent marginal counts by dots. For example, *n*_*j*,⋅,*k*_ represents the number of customers in restaurant *j* eating dish *k*; *m*_*j*,⋅_ represents the number of tables in restaurant *j*, and so on. At each table in each restaurant, one dish from the menu is ordered by the first customer at that table and shared by the remaining customers sitting at the same table.

A customer entering a restaurant can either choose an occupied table according to a probability proportional to the number of customers already seated at the table, or get a new table with a probability determined by the concentration parameter *α*. Specifically, in restaurant *j*, the *i*th customer chooses a dish (and thereby a table) according to

$$\begin{array}{c} \theta_{j,i}|\theta_{j,1},\cdots ,\theta_{j,i-1},\alpha ,G_{0}∼\;\sum_{t=1}^{m_{j,\cdot }}\;\frac{n_{j,t,\cdot }}{i-1+\alpha }\delta_{\psi_{j,t}}+\frac{\alpha }{i-1+\alpha }G_{0}, \end{array}$$ (3)

where *δ*_*ψ*_*j*,*t*__ is probability measure concentrated at *ψ*_*j*,*t*_. If a customer chooses an existing table, say *t*, then we increment *n*_*j*,*t*_ by one, and set *θ*_*j*,*i*_ = *ψ*_*j*,*t*_, *t*_*j*,*i*_ = *t*, and *z*_*j*,*i*_ = *k*_*j*,*t*_. If a new table is chosen, then we increment *m*_*j*,⋅_ by one, draw the dish for that table *ψ*_*j*,*m*_*j*,⋅_+1_ ∼ *G*_0_ and set *θ*_*ji*_ = *ψ*_*j*,*m*_*j*,⋅_+1_, *t*_*j*,*i*_ = *m*_*j*,⋅_ + 1, and *z*_*j*,*i*_ = *k*_*j*,*m*_*j*,⋅_+1_, where *k*_*j*,*m*_*j*,⋅_+1_ is the index of the drawn dish from *G*_0_.

Now let us consider the dish-level distributions. Similarly, a table can be served with an existing dish with probability proportional to the number of tables already serving the dish in the whole franchise, or with a new dish with probability determined by the concentration parameter *γ*. To be specific, the probability distribution of table *t* in restaurant *j* serving a particular dish is given by

$$\begin{array}{c} \psi_{j,t}|\psi_{1,1},\psi_{1,2},\cdots ,\psi_{2,1},\cdots ,\psi_{j,t-1},\gamma ,H∼\;\sum_{k=1}^{K}\;\frac{m_{\cdot ,k}}{m_{\cdot ,\cdot }+\gamma }\delta_{\phi_{k}}+\frac{\gamma }{m_{\cdot ,\cdot }+\gamma }H. \end{array}$$ (4)

If an existing dish is served, i.e., *k*_*j*,*t*_ ∈ {1, 2, ⋯, *K*}, we increment the count of that dish, *m*_⋅,*k*_*j*,*t*__, by one, and set *ψ*_*j*,*t*_ = *ϕ*_*k*_*j*,*t*__. If we choose a new dish, then we increment *K* by one. We also draw the new dish by *ϕ*_*K*+1_ ∼ *H*, and set *k*_*j*,*t*_ = *K* + 1.

This completes the description of the CRF metaphor. We summarize the variables, their meanings and how they relate to our problem in Table 1. We reiterate that the dishes are shared among the restaurants, which corresponds to a key property of the HDP.

**Table 1:** Meaning of the variables of the HDP process and their relationship to the FHR classification problem.

Variable	meaning
*x*_*j*,*i*_	served dish to the *i*th customer in the *j*th restaurant (observed features in the (*j*, *i*)th segment, i.e., in the *i*th segment of the *j*th recording)
*z*_*j*,*i*_	index of dish type served to the *i*th customer in the *j*th restaurant (index of the parameter of the (*j*, *i*)th segment)
*ϕ*_*k*_	*k*th dish type from the global menu (the *k*th parameter from the global set of parameters)
*ψ*_*j*,*t*_	dish type served on the *t*th table in the *j*th restaurant, (parameter of segments of the *j*th recording that belongs to the *t*th group)
*θ*_*j*,*i*_	dish type served to the *i*th customer in the *j*th restaurant (parameter of the (*j*, *i*)th segment)
*t*_*j*,*i*_	index of the table assigned to the *i*th customer in the *j*th restaurant (index of the group assigned to the (*j*, *i*)th segment)
*k*_*j*,*t*_	index of the dish type served on the *t*th table in the *j*th restaurant (index of the parameter of the *t*th group of the *j*th recording)

The HDP mixture model is a non-parametric Bayesian approach to data processing. It aims at modeling grouped data jointly, where each group (segment features of an FHR recording) is associated with a mixture model, and all the mixing components are shared across the groups (different FHR recordings share features). We assume that each dish type *ϕ*_*k*_ defines a mixing component that is used for generating actual dishes (features). We denote the generating distribution of the features by *F*(*ϕ*_*k*_). In summary, each observed feature *x*_*j*,*i*_ (the features of segment *i* of recording *j*) is generated by

$$\begin{array}{c} x_{j,i}∼F\left(\phi_{z_{j,i}}\right), \end{array}$$ (5)

where *ϕ*_*z*_*j*,*i*__ is the parameter of the feature distribution, and *z*_*j*,*i*_ is the index that defines the parameter. By setting the *F*’s to be Gaussian distributions, we obtain a Gaussian mixture model with HDP as the prior.

#### Chinese restaurant franchise with finite capacity

Now we consider a modified version of the CRF that was proposed in [18]. Assume that each restaurant has a limited capacity of accommodating customers, and without loss of generality, we assume that it is *N* for all the restaurants. Before the number of customers reaches that limit, the process is the same as in the CRF metaphor. After a restaurant is “full,” a new customer can come in and be seated only after the “oldest” customer leaves the restaurant. Then, the *i*th customer in the *j*th restaurant, where *i* > *N*, chooses a dish by

$$\begin{array}{c} \theta_{j,i}|\theta_{j,1},\cdots ,\theta_{j,i-1},\alpha ,G_{0}∼\;\sum_{t=1}^{m_{j,\cdot }^{*}}\;\frac{n_{j,t,\cdot }^{*}}{N-1+\alpha }\delta_{\psi_{j,t}}+\frac{\alpha }{N-1+\alpha }G_{0}, \end{array}$$ (6)

where the * notation represents the changes after the oldest customer (the (*i* − *N*)th of restaurant *j*) leaves. Similarly, we update the table counts after the table is chosen. In addition, the probability that table *t* in restaurant *j* serves a particular dish type is

$$\begin{array}{c} \psi_{j,t}|\psi_{1,1},\psi_{1,2},\ldots ,\psi_{2,1},\ldots ,\psi_{j,t-1},\gamma ,H∼\;\sum_{k=1}^{K^{*}}\;\frac{m_{\cdot ,k}^{*}}{m_{\cdot ,\cdot }^{*}+\gamma }\delta_{\phi_{k}}+\frac{\gamma }{m_{\cdot ,\cdot }^{*}+\gamma }H. \end{array}$$ (7)

After the dish is selected, the dish counts are updated accordingly.

We call this new process “Chinese restaurant franchise with finite capacity” (CRFC). The CRFC is designed to model grouped time-varying data, and capture the underlying dynamics. Simulation results on how the CRFC mixture model finds the cluster assignments of data over time can be found in [17].

### Inference

We describe a Markov chain Monte Carlo (MCMC) sampling scheme for estimating the parameters of the HDP and CRFC mixture models. This is a Gibbs sampling scheme based on the CRF [13]. To simplify the inference, the base distribution *H* is assumed to be conjugate to the data distribution *F*. For the non-conjugate case, the sampling approach can be adapted from techniques developed for non-conjugate DP mixtures [19]. In addition, here we assume known values for the concentration parameters *α* and *γ*. When they are unknown, we describe a sampling scheme for them in a later section. In the sequel, the notation ***x***^−*ij*^ signifies ***x*** = (*x*_*j*′*i*′_: all *j*′*i*′ except *j*, *i*), i.e., ***x***^−*j*,*i*^ = ***x***\*x*_*j*,*i*_. Similarly, ***t***^−*i*,*j*^ = ***t***\*t*_*j*,*i*_ and ***k***^−*j*,*t*^ = ***k***\*k*_*j*,*t*_. To make the sampling more efficient, instead of directly dealing with the *x*_*j*,*i*_s and *z*_*j*,*t*_s, we sample their indicator variables *t*_*j*,*i*_ and *k*_*j*,*t*_. We first describe the sampling of **t** and then the sampling of ***k***.

#### Sampling t

The prior probability of *t*_*j*,*i*_ taking an occupied table is proportional to $n_{j,t,\cdot }^{-j,i}$ according to Eq (3), where, as before, the notation ^−*j*,*i*^ means the corresponding variable is removed from a set or a count. And the prior probability of *t*_*j*,*i*_ taking a new value is proportional to *α*. More specifically, *t*_*j*,*i*_ is sampled from

$$\begin{array}{c} p\left(t_{j,i}=t|t^{-j,i},k\right)\propto \{\begin{array}{c} n_{j,t,\cdot }^{-j,i}f_{k_{j,t}}^{-x_{j,i}}\left(x_{j,i}\right),\;\;\;\;\;\text{if}\;t\;\text{is}\;\text{previously}\;\text{used}\\ \alpha p(x_{j,i}|t^{-j,i},t_{j,i}=t^{\text{new}},k),\;\text{if}\;t\;\text{is}\;\text{new} \end{array}, \end{array}$$ (8)

where $f_{k_{j,t}}^{-x_{j,i}}\left(x_{j,i}\right)$ represents the likelihood of sample *x*_*j*,*i*_ belonging to an existing mixture component *k*_*j*,*t*_ given all the other data, and is given by

$$\begin{array}{c} f_{k_{j,t}}^{-x_{j,i}}\left(x_{j,i}\right)=\frac{\int \;f\left(x_{j,i}|\phi_{k_{j,t}}\right)\;\prod_{\overset{j^{\prime },i^{\prime }\neq j,i}{z_{j^{\prime },i^{\prime }}=k_{j,t}}}\;f\left(x_{j^{\prime },i^{\prime }}|\phi_{k_{j,t}}\right)h\left(\phi_{k_{j,t}}\right)d\phi_{k_{j,t}}}{\int \;\prod_{\overset{j^{\prime },i^{\prime }\neq j,i}{z_{j^{\prime },i^{\prime }}=k_{j,t}}}\;f\left(x_{j^{\prime },i^{\prime }}|\phi_{k_{j,t}}\right)h\left(\phi_{k_{j,t}}\right)d\phi_{k_{j,t}}}. \end{array}$$ (9)

If a new table is chosen, i.e., *t*_*j*,*i*_ = *t*^*new*^, we need to draw a dish *k*_*j*,*t*^*new*^_ for *t*^*new*^, and the probability is

$$\begin{array}{c} p\left(k_{j,t^{new}}=k|t,k^{-j,t^{new}}\right)\propto \{\begin{array}{c} m_{\cdot ,k}f_{k}^{-x_{j,i}}\left(x_{j,i}\right)\;\text{if}\;k\;\text{is}\;\text{previously}\;\text{used}\\ \gamma f_{k^{new}}^{-x_{j,i}}\left(x_{j,i}\right)\;\;\;\text{if}\;k=k^{new}, \end{array} \end{array}$$ (10)

where

$$\begin{array}{ccc} f_{k^{\text{new}}}^{-x_{j,i}}\left(x_{j,i}\right) & = & \int \;f\left(x_{j,i}|\phi \right)h\left(\phi \right)d\phi \end{array}$$ (11)

is the prior density of *x*_*j*,*i*_. Therefore, the likelihood of a customer choosing a new table is

$$\begin{array}{c} p\left(x_{j,i}|t^{-j,i},t_{j,i}=t^{\text{new}},k\right)=\sum_{k=1}^{K}\;\frac{m_{\cdot ,k}}{m_{\cdot ,\cdot }+\gamma }f_{k}^{-x_{j,i}}\left(x_{j,i}\right)+\frac{\gamma }{m_{\cdot ,\cdot }+\gamma }f_{k^{\text{new}}}^{-x_{j,i}}\left(x_{j,i}\right). \end{array}$$ (12)

During sampling, some *n*_*jt*._ may become zero, i.e., the corresponding table *t* may become unoccupied. Then we need to update the corresponding dish count *m*_⋅,*k*_, which may result in deleting some mixture component if *m*_⋅,*k*_ = 0.

#### Sampling k

The likelihood of setting *k*_*j*,*t*_ = *k* is given by $f_{k}^{-x_{j,t}}\left(x_{j,t}\right)$, where ***x***_*j*,*t*_ represents all the *x*_*j*,*i*_s such that *t*_*j*,*i*_ = *t*, and $f_{k}^{-x_{j,t}}\left(x_{j,t}\right)$ is the conditional density of ***x***_*j*,*t*_ given all the data related to component *k* without ***x***_*j*,*t*_. For the conditional probability of *k*_*j*,*t*_, we can write

$$\begin{array}{c} p\left(k_{j,t}=k|t,k^{-j,t}\right)\propto \{\begin{array}{c} m_{\cdot ,k}^{-j,t}f_{k}^{-x_{j,t}}\left(x_{j,t}\right),\;\text{if}\;k\;\text{is}\;\text{previously}\;\text{used}\\ \gamma f_{k^{new}}^{-x_{j,t}}\left(x_{j,t}\right),\;\;\text{if}\;k=k^{new} \end{array}. \end{array}$$ (13)

The inference of CRFC mixture models can easily be obtained by changing the prior probabilities of the indicator variables *t*_*j*,*i*_ and *k*_*j*,*t*_.

## Features for classification

Here we present the complete list of features of FHR traces that we used for classification. As mentioned before, feature extraction has attracted much attention in the field of FHR analysis. The features can roughly be divided into three categories: time domain, frequency domain, and non-linear features. Time-domain features measure the variability of FHR signals in various forms, whereas the frequency domain features usually describe the powers in different frequency bands. Non-linear features quantify the non-linearity of FHR, e.g., with entropy and fractal dimension.

In our experiments, we divided the FHR series into non-overlapping segments, with length ranging from 40 to 120 samples. Then, from each segment we extracted one feature vector. This vector did not contain nonlinear features. Instead, it had 9 features from the time domain, and five from the frequency domain. The reason for not including non-linear features is that their reliable estimation usually requires much longer segments [9]. For example, the approximate entropy is applicable when the data series are longer than 100 samples [20].

In summary, we used only linear features from the time and frequency domains that are known from the literature on fetal heart rate processing. In the classification, we used 14 features, which are described in the next two subsections. However, the classifier operated in a feature space with reduced dimension and obtained via principle component analysis (PCA), as explained in the next section.

### Time-domain features

They include the mean and the standard deviation of the segment ***s***_*ji*_. In addition, we also use the short-term variability (STV) and long-term variability (LTV), which are defined in [8] as

$$\begin{array}{c} v_{STV}=\frac{1}{K}\;\sum_{k=1}^{K}\;\left|s\left(k+1\right)-s\left(k\right)\right|, \end{array}$$ (14)

$$\begin{array}{c} v_{LTV}=\frac{1}{M}\;\sum_{m=1}^{M}\;[\max_{k\in m}\left(s\left(k\right)\right)-\min_{k\in m}\left(s\left(k\right)\right)], \end{array}$$ (15)

where *s*(*k*), *k* = 1, …, *K* represents one segment of FHR series, *K* is the number of samples in each segment and *M* is the number of minutes of the segment. STV and LTV essentially quantify the changes of FHR series in different forms.

On the feature list, we also have the short-term irregularity (STI) and long-term irregularity (LTI) from [21] and defined by

$$\begin{array}{c} v_{STI}=\text{IQR}(\arctan\frac{s(k+1)}{s(k)}), \end{array}$$ (16)

$$\begin{array}{c} v_{LTI}=\text{IQR}(\sqrt{s^{2}\left(k\right)+s^{2}\left(k+1\right)}), \end{array}$$ (17)

where IQR stands for inter-quartile range with *k* = 1, …, *K*. In essence, STI and LTI describe the variability of FHR series too.

The other features are the standard descriptors of the Poincaré plot, SD1 and SD2, as well as the complex correlation measure (CCM) proposed in [22], which are defined by

$$\begin{array}{c} SD1^{2}=\gamma_{RR}\left(0\right)-\gamma_{RR}\left(1\right) \end{array}$$ (18)

$$\begin{array}{c} SD2^{2}=\gamma_{RR}\left(0\right)+\gamma_{RR}\left(1\right)-2{\bar{RR}}^{2} \end{array}$$ (19)

where *γ*_*RR*_(0) and *γ*_*RR*_(1) are the autocorrelation functions for lags 0 and 1 of the RR intervals, and $\bar{RR}$ being the mean of the RR intervals. The RR intervals are another representation of FHR, which stands for beat-to-beat interval and that can be obtained by

$$\begin{array}{c} \text{RR}=\frac{1}{\text{FHR}/60}. \end{array}$$ (20)

CCM, on the other hand, is a function of several lags of the autocorrelation functions of the RR intervals, or more specifically,

$$\begin{array}{c} CCM\left(m\right)=\frac{\gamma_{RR}\left(m-2\right)-2\gamma_{RR}\left(m-1\right)+2\gamma_{RR}\left(m+1\right)-\gamma_{RR}\left(m+2\right)}{2C_{n}\left(N-2\right)}, \end{array}$$ (21)

where *C*_*n*_ is a normalizing constant, defined as *C*_*n*_ = *π* × *SD*1 × *SD*2, and *m* is an integer. In our experiments, we set *m* = 1. These features are different types of descriptors of FHR variability.

### Frequency-domain features

These features represent powers in four frequency bands: very low frequency (VLF: 0–0.06 Hz), low frequency (LF: 0.06–0.3 Hz), medium frequency (MF: 0.3–1 Hz) and high frequency (HF: 1–2 Hz). In addition, they also include the ratio of powers of two bands LF/(MF+HF). The frequency-domain features represent the underlying physiological activity of either the mother or the fetus. It is worth noting that there is no consensus on how to define the frequency bands. In our experiments, we used the ranges from [23].

The complete list of features is shown in Table 2.

**Table 2:** List of all the features.

Category	Feature
	Mean, standard deviation
Time	STV, STI, LTV, LTI, SD1, SD2, CCM
Frequency	VLF, LF, MF, HF, ratio

## Experimental settings

In this section, we describe in detail our experiments of classifying FHR signals using non-parametric Bayesian models.

### Database

In our work, we used the open-access cardiotocography (CTG) database collected from the Czech Technical University (CTU) and University Hospital in Brno (UHB) [24]. This database contains 552 CTG recordings, each comprising an FHR time tracing and a uterine contraction (UC) signal, both sampled at 4 Hz. All recordings start at a maximum of 90 minutes before delivery. Fetal outcome data, which include measurements of umbilical artery blood samples and Apgar scores evaluated at 1 and 5 minutes after delivery, are available for assessment purposes. Additional fetal and delivery information, such as sex, weight, type of delivery, are also collected. More details on the data collection can be found in [25].

### Pre-processing and segmentation

The acquisition of FHR signals suffers from different kinds of artifacts, which are generally caused by maternal and fetal movements or displacements of the transducer used in the acquisition. There are two types of artifacts, either the measured samples are incorrect or they are simply missing (the values are equal to 0). Therefore, the FHR signals have to be pre-processed before they are used for analysis.

**Fig 1:**
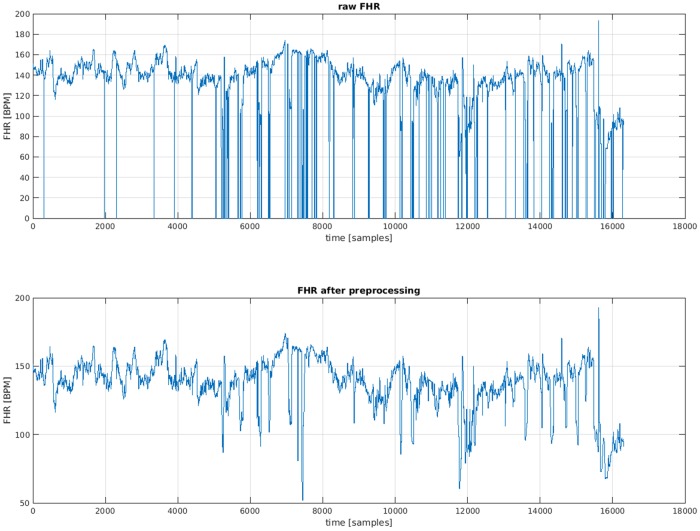
Comparison between a raw FHR signal and the signal obtained after pre-processing.

In practice, any successive samples with differences greater than 25 bpm are considered as artifacts. All artifacts, including missing data with duration less than 15 seconds, are interpolated by piecewise cubic Hermite polynomial method. If the duration is longer than 15 seconds, they are simply discarded. Fig 1 shows an example of an FHR series before and after pre-processing.

Out of 552 FHR recordings, we selected a balanced dataset with the same number of recordings and labeled as *healthy* and *unhealthy*. The labels were defined by the following criteria: an FHR recording is healthy if its associated umbilical cord pH value is greater than a threshold *τ*_0_, and it is labeled as unhealthy if the pH value is less than or equal to *τ*_1_. There is no consensus on the exact values of the thresholds, so we experimented with *τ*_0_ = 7.2 and both *τ*_1_ = 7.05 as in [9] and *τ*_1_ = 7.1 as in [10]. The number of recordings *N* in the selected dataset ended up with 88 and 122 respectively.

In our experiments, the last *M*-minute data of the FHR recordings were analyzed. Each recording was divided into non-overlapping segments of *l* seconds, where *l* ranged from 10 to 30 seconds. Thus, the number of segments in each series was *m*, where *m* = *M* × 60/*l*. For each segment ***s***_*ji*_, which is the *i*-th segment in the *j*-th recording, a feature vector ***x***_*ji*_ of dimension *d* was extracted.

### Dimensionality reduction

As described in Section 1, in our experiments, each feature vector has 14 dimensions. High dimensionality is usually difficult to deal with, specifically in terms of issues such as computational costs and convergence in Gibbs sampling. Hence, before training the models, we reduced the dimension of the feature space from 14 to *q* by way of principle component analysis (PCA) [26].

**Fig 2:**
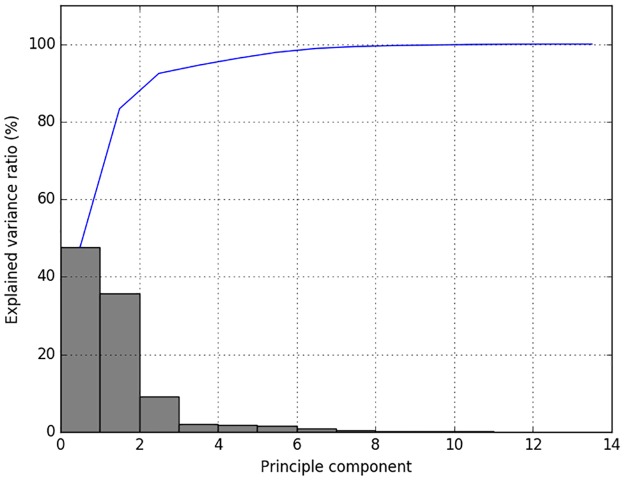
Explained variance ratio as a function of number of principle components.

Since PCA is sensitive to the scales of different dimensions of input data and the ranges of feature values in each dimension can vary largely, we scaled these values into the interval (−1, 1) before applying PCA. After scaling, we computed the variance ratio of each component. An example of PCA results of all the data when the number of recordings *N* = 88 and the segment length *l* = 10 is shown in Fig 2. The gray bars are the explained variance ratios of each principle component, and the blue line represents the cumulative variance ratio.

According to the preliminary analysis of all the data, we concluded that most of the variance lies in the first 4 principle components. Therefore, we experimented with different choices of *q* = 2, 3, and 4. Note that in each iteration of cross-validation, only the training data were used to obtain the linear transformation matrix, and the testing data were transformed accordingly.

### Model priors

The HDP and CRFC mixture model both have two concentration parameters, *γ* and *α*, as described in Section 1. Instead of assigning fixed values to them, we implemented an auxiliary sampler provided in [13] to infer them. In our experiments, the concentration parameters were given gamma priors, *γ* ∼ gamma(1, 1) and *α* ∼ gamma(10, 1). Therefore, in our experiments, we needed to choose only two variables: the segment length *l* and the feature dimension *q* after PCA.

### Classification process

The process of using HDP based models to classify FHR tracings is as follows. The last 30-minute data were used in the classification tasks. During the training stage, two HDP Gaussian mixture models (HDPGMs), $M_{0}$ and $M_{1}$, were constructed from the FHR recordings and labeled as healthy and unhealthy, respectively. For estimation of the models’ parameters, we implemented the collapsed Gibbs sampler (proposed in [13]). During the testing stage, given a new FHR tracing ***x***_*j*_, the classification is made by comparing the likelihoods *L*_0_ and *L*_1_, which are defined by

$$\begin{array}{c} \begin{array}{cc} & L_{0}=e^{l_{0}},\;L_{1}=e^{l_{1}},\\ & l_{0}=\log f\left(x_{j}|M_{0}\right)=\sum_{i=1}^{m}\log f\left(x_{ji}|M_{0}\right),\\ & l_{1}=\log f\left(x_{j}|M_{1}\right)=\sum_{i=1}^{m}\log f\left(x_{ji}|M_{1}\right). \end{array} \end{array}$$ (22)

If *L*_0_ > *L*_1_, the FHR series is classified as healthy and vice versa. Note that here we assume that the priors of the fetuses were equal.

In using the CRFC Gaussian mixture models, first we set a window length *M*_win_ equal to 30 minutes. Essentially, this is equivalent to the restaurant capacity in the CRF metaphor. We analyzed the last 45 minutes of FHR recordings. Two models, $M_{0}$ and $M_{1}$ were initiated from the first 30-minute data (i.e., the last 45 to 15 minutes from the original FHR series) from the respective groups. At each time instance, we moved the window by one segment, and trained the models by adding new data and removing the oldest data. The likelihoods of being healthy and unhealthy, *L*_0_ and *L*_1_, were computed similarly to (22).

We define the probabilities of FHR series corresponding to healthy or unhealthy fetuses, denoted as *p*_0_ and *p*_1_, by

$$\begin{array}{c} \begin{array}{cc} & p_{0}=\frac{e^{l_{0}/m}}{e^{l_{0}/m}+e^{l_{1}/m}}\\ & p_{1}=1-p_{0} \end{array} \end{array}$$ (23)

where *m* is the number of segments in each FHR series. We call this method the “naïve approach”. A modified version of the probabilities is defined as follows.

$$\begin{array}{c} \begin{array}{cc} & l_{0}^{\prime }=l_{0}^{\left(1\right)}\;\cdot \;w_{1}+\cdots +l_{0}^{\left(m\right)}\;\cdot \;w_{m},\\ & l_{1}^{\prime }=l_{1}^{\left(1\right)}\;\cdot \;w_{1}+\cdots +l_{1}^{\left(m\right)}\;\cdot \;w_{m},\\ & p_{0}^{\prime }=\frac{e^{l_{0}^{\prime }}}{e^{l_{0}^{\prime }}+e^{l_{1}^{\prime }}},\;p_{1}^{\prime }=1-p_{0}^{\prime } \end{array} \end{array}$$ (24)

where

$$\begin{array}{c} l_{0}^{\left(i\right)}=\log f\left(x_{ji}|M_{0}\right),\;l_{1}^{\left(i\right)}=\log f\left(x_{ji}|M_{1}\right) \end{array}$$ (25)

and *w*_*i*_’s are weights defined as

$$\begin{array}{c} w_{i}=u_{i}/\sum_{i=1}^{m}\;u_{i} \end{array}$$ (26)

where *u*_*i*_ is the percentage of data that are not interpolated in the *i*-th segment, which is a measure of signal quality. We call this method the “weighted approach”.

### Performance assessment

We assessed the classification performance of the models with the standard metrics, true positive rate (TPR) and true negative rate (TNR). We also used the weighted relative accuracy (WRA) [27], which is defined by WRA = 4 × cost × (TPR − FPR)/(1 + cost)^2^, where FPR represents false positive rate. In this study, we assigned the cost to 1.

To fully utilize the dataset and avoid the bias caused by randomly selecting training/testing data, we used the 5-fold cross-validation (CV) method for performance assessment. At each iteration, 80% of the data were used for training and the rest for testing. The outcome metrics were averaged across all iterations and the mean values were reported.

## Results

In this section, we first provide the classification performance of HDPGMs and the comparison with that of SVMs, which achieved the best performance in studies [9, 12]. Then we show the real-time classification of FHR tracings by models based on CRFC.

### Performance by HDPGMs

As described in Section 1, we experimented with two different thresholds *τ*_1_ that delineate the non-healthy group of fetuses. By setting *τ*_1_ = 7.05, the number of recordings with total length exceeding 30 minutes *N* is 88, and for *τ*_1_ = 7.1, *N* equals 122. After segmentation, feature extraction and PCA, the dataset was transformed to *N* groups of data, each group containing *m* observations of dimension *q*. We experimented with different choices of segment length *l* and dimension *q*. The results, with the best performance highlighted in bold font, are provided in Table 3,.

**Table 3:** Performance of HDPGMs.

N	*q*	*l*	TPR	TNR	WRA
88	2	**10 sec**	**0.753**	**0.844**	**0.597**
		20 sec	0.708	0.822	0.531
		30 sec	0.681	0.844	0.525
	3	10 sec	0.706	0.800	0.506
		20 sec	0.636	0.844	0.480
		30 sec	0.655	0.867	0.522
	4	10 sec	0.700	0.733	0.433
		20 sec	0.656	0.800	0.456
		30 sec	0.642	0.703	0.344
122	2	10 sec	0.637	0.753	0.390
		20 sec	0.606	0.769	0.376
		**30 sec**	**0.654**	**0.754**	**0.408**
	3	10 sec	0.654	0.704	0.358
		20 sec	0.655	0.721	0.376
		30 sec	0.554	0.803	0.356
	4	10 sec	0.622	0.720	0.342
		20 sec	0.604	0.738	0.342
		30 sec	0.587	0.719	0.306

The same datasets were used to test the SVM-based method. The classification process was as follows: instead of segmentation, the 14 features were extracted from the whole FHR series of the last 30 minutes. The feature vectors were scaled to the range (−1, 1), and then used as input to the SVMs classifier. The SVMs classification algorithms had two free parameters: cost *C* and *γ*. We searched for the optimal combination of these parameters in terms of the testing performance metric, WRA. Five-fold CV method was used to eliminate biases. The results obtained by SVMs are shown in Table 4.

**Table 4:** Peformance of SVMs.

N	C	*γ*	TPR	TNR	WRA
88	3	0.1	0.650	0.867	0.517
122	1	0.1	0.556	0.836	0.392

By comparing the results in Tables 3 and 4, we conclude that in both cases of *τ*_1_, the proposed method outperformed the SVM-based method.

### Real-time classification

**Fig 3:**
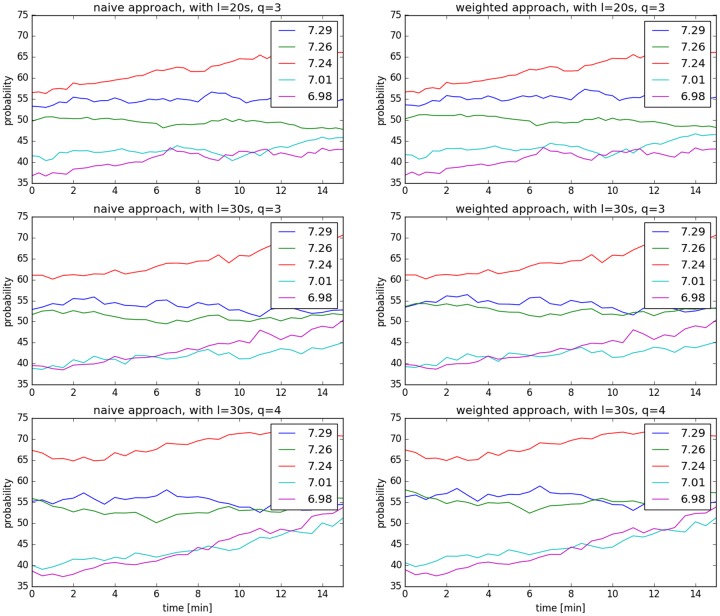
One instance of real-time classification results. The X axis represents time and the Y axis represents probability.

In this experiment, we set the threshold *τ*_1_ = 7.05. The number of FHR recordings of total lengths greater than 45 minutes is 70. We randomly chose 60 recordings for training the CRFC Gaussian mixture models, and the rest for testing. Due to lack of labels of FHR recordings at each time instant, we assumed that the training series stayed in the same group for the whole duration. At each time instant, we computed the probability of the FHR series being associated with a healthy fetus. We used both, the naïve and the weighted methods. Fig 3 shows the changes of probabilities of different FHR recordings being healthy over time. The corresponding pH values are given in the legends. The left three figures are the probabilities obtained by the naïve approach and the right are obtained by the weighted approach. The figures in the different rows correspond to different experimental settings.

From the results, we can observe small differences between the two approaches. The probabilities obtained by different experimental settings are not identical but agree with each other in terms of the overall trend.

## Conclusion

In this paper, we implemented the hierarchical Dirichlet process mixture model and its variation in classifying fetal heart rate tracings. In our method, we employed 14 features that have been used in the literature before. We showed that our method outperformed the state-of-the-art algorithm in terms of weighted relative accuracy when using the same feature set. Furthermore, we demonstrated how our method can be adapted to online learning of data and computing the probability of a fetus being healthy in real-time.

The merits of non-parametric Bayesian models, as shown in our experiments, are being free from parameter-tuning and model selection. In addition, the experiment results suggested that our methods were able to accurately model the FHR data. On the other hand, the Chinese restaurant franchise with finite capacity models are able to process data of a fixed length sequentially. Therefore, if applied in real-world scenarios, the CRFC model can evaluate the FHR data with time and provide the physicians with real-time estimates of the fetal status. However, in our experiments, since the true online fetal health information was unavailable, we were unable to validate how our method performed.
